# Analysis of Risk Factors for Leukoaraiosis: A Multicenter Retrospective Study

**DOI:** 10.1002/brb3.71006

**Published:** 2025-10-29

**Authors:** Lingqi Sun, Wang Guo, Yi Li, Li Fang, Ji Ma, Xiangdong Tang

**Affiliations:** ^1^ Sleep Medicine Center, Mental Health Center West China Hospital, Sichuan University Chengdu Sichuan People's Republic of China; ^2^ Southwest Jiaotong University Chengdu Sichuan People's Republic of China; ^3^ Department of Ultrasound Medicine Air Force Hospital of the Western Theater of the Chinese People's Liberation Army Chengdu Sichuan People's Republic of China; ^4^ Department of Otolaryngology Air Force Hospital of the Western Theater of the Chinese People's Liberation Army Chengdu Sichuan People's Republic of China; ^5^ Department of Medical Oncology West China Hospital Sichuan University Chengdu Sichuan People's Republic of China

**Keywords:** leukoaraiosis, risk factors, retrospective study, ROC curve

## Abstract

**Background and Aim:**

Leukoaraiosis is a common imaging marker of cerebral small vessel disease. There is now increasing evidence shows the relationship between leukoaraiosis and cognitive impairment, high risk of death after stroke. The aim of this study was to analyze the risk factors clinically associated with the development of leukoaraiosis, and to explore clinical biomarkers that may predict leukoaraiosis.

**Methods:**

Inpatients were continuously recruited from July 2014 to October 2020. After admission, the cranial MRI examination was evaluated, and the severity of leukoaraiosis were evaluated and graded. Vascular risk factors and relevant clinical data were collected. Univariate analysis was used to analyze the parameters, and multivariate logistic regression analysis was used to analyze the statistically significant parameters. The analysis results were plotted as ROC curve to find out the diagnostic accuracy of the model.

**Results:**

1) 327 patients meeting the study criteria were included. Univariate analysis showed that 13 factors were statistically significantly (*p* < 0.05). 2) Multivariate logistic regression model showed that age (Age 1 [OR, 14.315; 95% CI, 6.662–30.757; *p* = 0.000], Age 2 [OR, 53.062; 95% CI, 15.661–179.783; *p* = 0.000]), elevated systolic blood pressure (SBP 1 (OR, 2.927; 95% CI, 1.224–7.003; *p* = 0.016), SBP 3 (OR, 15.109; 95% CI, 1.380–165.385; *p* = 0.026)), ischemic stroke (OR, 5.990; 95% CI, 2.594–13.846; *p* = 0.000), and FT4 (OR, 4.836; 95% CI, 2.086–11.216; *p* = 0.000) were independent risk factors for leukoaraiosis. 3) The ROC curve indicated the accuracy of diagnosis on leukoaraiosis is 0.906, and the positive rate and negative rate are both 85.2%.

**Conclusions:**

1) Our findings support age, systolic blood pressure, ischemic stroke, and FT4 level serving as factors affecting the development of leukoaraiosis. 2) The model of “age, systolic blood pressure, ischemic stroke, FT4” may have relatively ideal sensitivity and specificity in predicting the development of leukoaraiosis.

## Graphical abstract

This study was to analyze the risk factors clinically associated with the development of leukoaraiosis, and to explore clinical biomarkers that may predict leukoaraiosis. The results demonstrate that age, systolic blood pressure, ischemic stroke, and FT4 level serving as factors affecting the development of leukoaraiosis.

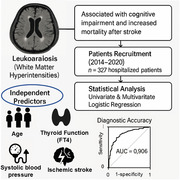

**Abbreviations**
95% CI: 95% confidence intervals
ALT: alanine aminotransferase
AST: aspartate aminotransferase
AUC: area under the curve
BBB: blood–brain barrier
BMI: body mass index
CA199: glycoconjugate antigen 199
CAHD: coronary atherosclerotic heart disease
CEA: carcinoembryonic antigen
cfPWV: carotid‐femoral pulse wave velocity
CSVD: cerebral small vessel disease
CT: computed tomography
CTA: computer tomography angiography
DBIL: direct bilirubin
DBP: diastolic blood pressure
DSA: digital subtraction angiography
DWI: diffusion‐weighted imaging
FBG: fasting blood glucose
FIB: fibrinogen
FLAIR: fluid‐attenuated inversion recovery sequence
fMRI: functional MRI
FT3: free triiodothyronine
FT4: free thyroxine
HbA1c: glycosylated hemoglobin
HDL: high density lipoprotein cholesterol
IBIL: indirect bilirubin
LA: leukoaraiosis
LDL: low density lipoprotein cholesterol
MRA: magnetic resonance angiography
MRI: magnetic resonance imaging
ORs: dominance ratios
ROC: receiver operating characteristic
SBP: systolic blood pressure
T1WI: T1‐weighted imaging
T2WI: T2‐weighted imaging
TBIL: total bilirubin
TC: total cholesterol
TG: triglyceride
UA: uric acid
WMH: white matter hyperintensity
WMLs: white matter lesions

## 1 Background

Leukoaraiosis (LA), also known as white matter hyperintensity (WMH) or white matter lesions (WMLs), is an imaging marker of cerebral small vessel disease (CSVD). LA lesions, which are multifocal or diffuse with indistinct borders, are most commonly located near the cerebral ventricles or within the centrum semioval. On computed tomography (CT) images, they appear hypodense compare to normal white matter. Magnetic resonance imaging (MRI) is more clear, showing T2‐weighted (T2WI) and/or fluid‐attenuated inversion recovery sequences (FLAIR) with hyperintensity and T1‐weighted imaging (T1WI) with isointensity or hypointensity, without cystic lesion (Sun et al. 2022).

LA, is characterized pathologically by pale myelin, demyelination, oligodendrocyte apoptosis, and vacuole formation (Hannawi 2024). Although LA is gaining widespread attention, its pathogenesis remains unclear. It might be associated with cerebral blood flow autoregulation, venous collagen deposition, blood–brain barrier (BBB) disruption, and genetic factors (Lin et al. 2017). LA is common in the elderly, especially in elderly patients with vascular risk factors. In the general population, the prevalence of LA ranges from 11% to 21% around Age 64 to as high as 94% at Age 82 (Huang et al. 2024). Although the pathogenesis of LA is not fully understood, it is generally accepted that its incidence increases with age, especially in people over 60 years of age (Marek et al. 2018). Other risk factors include hypertension, female gender, abdominal obesity, history of stroke, hyperlipidemia, carotid stenosis, heart disease, chronic kidney disease, Type 2 diabetes, hyperhomocysteinemia, smoking and alcohol abuse, among others (Ben‐Assayag et al. 2012; Rościszewska‐Żukowska et al. 2013; Mijajlović et al. 2011; Helenius and Tatlisumak 2007). Controversial risk factors, including epilepsy, tumor markers, changes in thyroid function, etc., may be associated with the development of LA (Ferlazzo et al. 2016; Son et al. 2020; Leonards et al. 2014). Due to different research methods, some of the findings are inconsistent and even contradictory. However, to date, data about the risk factors of LA are limited.

Although LA has long been considered an incidental finding with no therapeutic necessity, there is evidence that LA is associated with specific clinical manifestations such as cognitive decline, gait impairment, mood disorders, urinary dysfunction, and disability recently (Wang et al. 2022; Grueter and Schulz 2012; Morley 2015; Cai et al. 2015). In lights of the clinical implications of LA, it may be important to explore surrogate markers for LA. Unfortunately, most of previous studies were performed in stroke patients, so the effect of stroke on the correlation could not be completely excluded. There also hasn't been any study that comprehensively incorporates the above risk factors into research. Therefore, predicting the risk of developing LA based on clinical features, and to minimizing adverse clinical outcomes, remains a fertile field for clinical research. In this study, we selected patients with pure and preclinical LA to investigate the risk factors of LA, and comprehensively included all possible risk factors related to LA in a multicenter retrospective study. The aim of this study was to analyze the clinical risk factors associated with the development of LA, to provide a simple and rapid possible method for early diagnosis of LA.

## 2 Materials and Methods

### 2.1 Subjects

Consecutive inpatients attending the Department of Neurology at Sichuan Provincial People's Hospital, General Hospital of the Western Theater of the Chinese people's Liberation Army, and Air Force Hospital of the Western Theater of the Chinese People's Liberation Army were included in this study from July 2014 to October 2020. The Department of Neurology was selected because LA is more common in this group. Patients here often have complex neurological conditions, such as cerebrovascular diseases and cognitive impairments, which are closely linked to LA. This made the department suitable for examining the risk factors and biomarkers associated with LA. To substantiate the choice of setting, an audit of our admission‐MRI screening log over this period identified LA in 611 of 1023 unique neurology inpatients (59.7%), indicating a higher inpatient prevalence than typically reported in community cohorts of comparable age and supporting the feasibility of biomarker discovery within a reasonable recruitment window (Lin et al. 2017; Lam et al. 2021, Huang et al. 2024)

Inclusion criteria were the following (Son et al. 2020; Park et al. 2012; Seo et al. 2019): age ≥ 18 years; liver function, renal function, blood glucose, blood lipid, thyroid function, tumor markers and cranial MRI, magnetic resonance angiography (MRA)/computer tomography angiography (CTA)/digital subtraction angiography (DSA), carotid ultrasound have been completed, and the imaging data are complete and clear. All scans were free from motion artifacts, properly oriented for consistent anatomical assessment, and captured at clinically appropriate resolutions with sufficient signal‐to‐noise ratio to clearly identify the regions of interest. Exclusion criteria were the following: Other diseases that may affect LA image analysis, including WMLs caused by hereditary, infectious, metabolic, immune, and toxic conditions; missing cranial MRI, MRA/CTA/DSA and other laboratory data; combined malignancy, severe acute and chronic liver disease, dementia. The above diseases were excluded as it could impact metabolic processes and confound both imaging and biochemical data. The protocol of this study was approved by Ethics Committee of Air Force Hospital of the Western Theater of the Chinese People's Liberation Army, the ethics code is 20210107007.

### 2.2 Clinical Data Collection

In selecting the clinical factors for analysis, we focused on those consistently reported in the literature as being associated with LA. These factors were chosen based on their strong biological plausibility, established relevance in clinical practice, and robust evidence from previous studies. All clinical data of the enrolled patients were obtained by two specialized neurologists, without knowledge of the patients' cranial imaging data, and the following information was recorded in detail: gender, age, body mass index (BMI), ischemic stroke, coronary atherosclerotic heart disease (CAHD), hypertension and blood pressure on admission, history of diabetes and hyperlipidemia, cranial and carotid ultrasound, epilepsy history and seizure type, history of smoking and alcohol consumption; laboratory indicators, including total bilirubin (TBIL), indirect bilirubin (IBIL), direct bilirubin (DBIL), aspartate aminotransferase (AST), alanine aminotransferase (ALT), total cholesterol (TC), triglyceride (TG), low density lipoprotein cholesterol (LDL), high density lipoprotein cholesterol (HDL), fibrinogen (FIB), glycosylated hemoglobin (HbA1c), fasting blood glucose (FBG), free triiodothyronine (FT3), free thyroxine (FT4), uric acid (UA), carcinoembryonic antigen (CEA), glycoconjugate antigen 199 (CA199), systolic blood pressure (SBP), diastolic blood pressure (DBP) on admission.

The relevant clinical indicators are defined as follows: BMI = weight (kg)/height^2^ (m^2^), where height and weight are measured on admission. History of ischemic stroke: previous definite diagnosis of ischemic stroke or definite imaging evidence on this admission clearly suggesting signs of infarction, including types of cerebral thrombosis and cerebral embolism. History of CAHD: previous definite diagnosis of CAHD or definite evidence of diagnosis by relevant ancillary tests on this admission. History of hypertension: previous or current admission measured SBP ≥ 140 mmHg and DBP ≥ 90 mmHg, or taking regular oral antihypertensive medication. History of diabetes: previous or current admission with a clear diagnosis of diabetes or taking regular oral hypoglycemic medication. History of hyperlipidemia: previous definite diagnosis of hyperlipidemia or taking regular oral lipid‐lowering medication, or this admission measured TC ≥ 5.72 mmol/L, TG ≥ 1.70 mmol/L, and LDL ≥ 3.10 mmol/L. Cranial and carotid ultrasound: previous definite diagnosis of intracranial or carotid artery stenosis, or this admission was completed with relevant ancillary tests suggesting vascular stenosis. Epilepsy history and seizure type: previous or current admission with a clear diagnosis of epilepsy, and a history of seizures or being on regular oral antiepileptic medication, with relevant tests suggesting abnormal epileptic discharges, 2017 International League Against Epilepsy (ILAE) for classification and definition of epilepsy (Fisher et al. 2017). History of smoking and alcohol consumption: history of regular smoking (≥ 10 cigarettes/day) and alcohol consumption (≥ 50 g/day) for ≥ 2 years is defined as a history of smoking/alcohol consumption (Table 1).

**TABLE 1:** Grouping scale and single factor comparison of clinical data of leukoaraiosis and non‐leukoaraiosis patients.

		LA	Non‐LA	*p* value
Factor	Total (*n* = 327)	(*n* = 156)	(*n* = 171)	
Population characteristics				
Male, *n* (%)	172 (52.6)	83 (53.2)	89 (52)	0.834
Age (years)	62 ± 12	71 ± 5.5	51 ± 7.75	0.000^*^
BMI	23.33 ± 3.3	23.4 ± 3.44	23.27 ± 3.18	0.427
Vascular risk factors				
Ischemic stroke, *n* (%)	178 (54.4)	115 (73.7)	63 (36.8)	0.000^*^
CAHD, *n* (%)	10 (3.1)	9 (5.8)	1 (0.6)	0.008^*^
Vascular stenosis, *n* (%)	76 (23.2)	50 (32.1)	26 (15.2)	0.000^*^
Hypertension, *n* (%)	135 (41.3)	87 (55.8)	48 (28.1)	0.000^*^
Diabetes, *n* (%)	67 (20.5)	34 (21.8)	33 (19.3)	0.576
Hyperlipidemia, *n* (%)	83 (25.4)	37 (23.7)	46 (26.9)	0.509
Smoking, *n* (%)	93 (28.4)	42 (26.9)	51 (29.8)	0.561
Drinking, *n* (%)	84 (25.7)	42 (26.9)	42 (24.6)	0.625
Clinical features				
Epilepsy	15 (4.6)	5 (3.2)	10 (5.8)	0.254
TBIL, umol/L	15.4 ± 4.1	15.5 ± 4.15	15.4 ± 4.03	0.869
DBIL, umol/L	4.4 ± 1.34	4.7 ± 1.45	4.3 ± 1.15	0.035^*^
IBIL, umol/L	10.8 ± 2.99	10.45 ± 3.46	11.1 ± 2.8	0.282
ALT, U/L	21.9 ± 9.43	21.75 ± 8.4	22 ± 9.76	0.641
AST, U/L	26 ± 6	27.15 ± 7	25 ± 5.93	0.012^*^
TC, mmol/L	4.39 ± 1.02	4.34 ± 1.07	4.43 ± 0.98	0.394
TG, mmol/L	1.19 ± 0.45	1.2 ± 0.35	1.19 ± 0.51	0.648
LDL, mmol/L	2.45 ± 0.52	2.36 ± 0.53	2.51 ± 0.5	0.080
HDL, mmol/L	1.19 ± 0.2	1.16 ± 1.19	1.21 ± 0.2	0.409
FIB, g/L	2.76 ± 0.47	2.89 ± 0.55	2.67 ± 0.44	0.009^*^
HbA1c, %	5.71 ± 0.38	5.8 ± 0.43	5.68 ± 0.35	0.053
FBG, mmol/L	5.2 ± 0.63	5.33 ± 0.82	5.1 ± 0.53	0.053
FT3, pmol/L	3.52 ± 0.7	3.18 ± 0.68	3.83 ± 0.69	0.000^*^
FT4, pmol/L	9.48 ± 5.53	1.33 ± 5.31	11.03 ± 5.59	0.000^*^
UA, umol/L	307 ± 60.93	308.5 ± 60.48	305 ± 58.98	0.720
CEA, ng/mL	1.78 ± 0.63	2.01 ± 0.69	1.55 ± 0.49	0.000^*^
CA199, U/mL	7.02 ± 4.9	7.9 ± 0.32	5.9 ± 4.12	0.004^*^
SBP, mmHg	130 ± 13.5	138 ± 14	126 ± 13	0.000^*^
DBP, mmHg	78 ± 9.5	80 ± 9.63	75 ± 8.25	0.111

Abbreviations: ALT, alanine aminotransferase; AST, aspartate aminotransferase; BMI, body mass index; CA199, glycoconjugate antigen 199; CAHD, coronary atherosclerotic heart disease; CEA, carcinoembryonic antigen; DBIL, direct bilirubin; DBP, diastolic blood pressure; FBG, fasting blood glucose; FIB, fibrinogen; FT3, free triiodothyronine; FT4, free thyroxine; HbA1c, glycosylated hemoglobin; HDL, high density lipoprotein cholesterol; IBIL, indirect bilirubin; LA, leukoaraiosis; LDL, low density lipoprotein cholesterol; SBP, systolic blood pressure; TBIL, total bilirubin; TC, total cholesterol; TG, triglyceride; UA, uric acid.

* represents p < 0.05, statistically different.

### 2.3 Brain MRI Examination and Grade of LA

**FIGURE 1:**
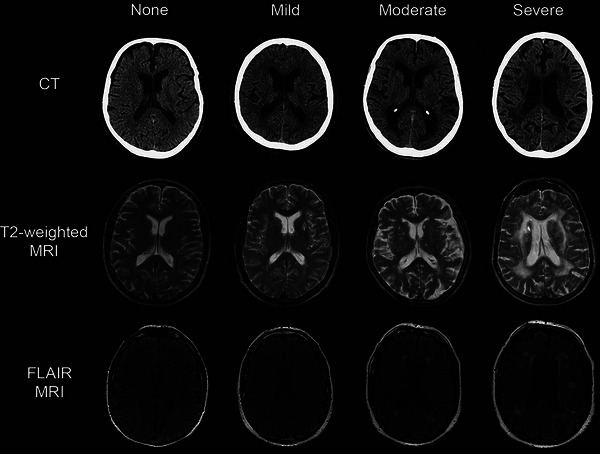
Imaging manifestations and grading of leukoaraiosis (Grueter and Schulz 2012).

**FIGURE 2:**
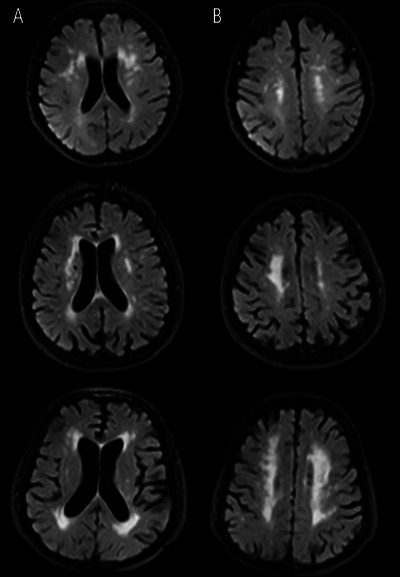
Leukoaraiosis severity grading. (A) The grading system for the periventricular matter, (B) The grading system for the deep white matter. Grades “1”, “2”, and “3” from top to bottom.

All enrolled patients underwent cranial MRI (1.5T‐3.0T). The MRI sequences include T1WI, T2WI, FLAIR, and diffusion‐weighted imaging (DWI). The presentation of LA on cranial MRI is bilaterally symmetrical distribution of white matter areas on T2WI and FLAIR with blurred borders and irregular high signal, isosignal or slightly low signal on T1WI, and isosignal on DWI, among which FLAIR is the most clearly visualized. The lesions within 1 cm of the lateral ventricular wall were considered as paraventricular LA, while the lesions outside 1 cm of the lateral ventricular wall were considered as deep LA, where lesions larger than 10 mm were considered as fusion lesions. According to Fazekas grading scale (Ben‐Assayag et al. 2012), the severity of LA were evaluated and graded (Figure 1). The grading system for the periventricular matter is as follows: 0—no lesions, 1—“caps” or pencil‐thin lining, 2—smooth “halo,” and 3—irregular periventricular signal extending into the deep white matter. The grading system for the deep white matter is as follows: 0—no lesions, 1—punctate foci, 2—beginning confluence, and 3—large, confluent areas (Figure 2).

### 2.4 Statistical Analysis

The data were analyzed using IBM SPSS 23.0 software. The enumeration data are expressed in number or percentage. Abnormal distribution measurement data are expressed using median ± interquartile range. Normal distribution measurement data are expressed as mean ± standard deviation. Prior to further analysis, we carefully evaluated the dataset for completeness. Although all patients underwent cranial MRI, a very small number of screened patients had missing values in some laboratory parameters, including thyroid function measures (FT3, FT4), HbA1c, and tumor markers (CEA, CA‐199). The missing rate for each of these variables was below 3%. These tests were not always performed in routine clinical practice when no specific indication was present. Patients with such missing values were excluded at the enrollment stage, according to our predefined criteria. As a result, the final analytical cohort contained no missing values for the variables used in our models. To ensure that excluding these patients did not bias the results, we compared baseline characteristics between included patients with complete data and those excluded due to missing data. No statistically significant differences were found between the groups (all *p* values > 0.05). This supports the assumption that the missingness was Missing Completely at Random (MCAR) and confirms that our complete‐case approach did not introduce systematic bias.

After confirming data integrity, we randomly split the dataset into a training set (80% of cases) and a testing set (20% of cases) using a stratified sampling approach to ensure balanced representation of key variables. Within the training set, univariate analyses were conducted using Chi‐square tests, Fisher's exact tests, *t*‐tests, or rank‐sum tests, depending on the data type. Statistically significant variables (*p* < 0.05) from these analyses were then included in multivariate logistic regression models. The results of these models were used to identify the most relevant risk factors, which were further assessed for potential interactions.

For model validation, we plotted receiver operating characteristic (ROC) curves based on the training set to determine the area under the curve (AUC), optimal cutoff points, and corresponding sensitivity and specificity values. These cutoff points and regression coefficients were subsequently applied to the testing set to evaluate the model's performance on unseen data. This approach allowed us to ensure that the model's diagnostic accuracy and sensitivity/specificity values were not simply the result of overfitting but were generalizable.

## 3 Results

### 3.1 Baseline Characteristics

A total of 327 patients were eventually included in our study. The mean age of the patients was 62 ± 12 (18–93) years, of which 172 (52.6%) were males and 155 (74.4%) were females. Among the patients, 171 (52.3%) did not have LA (LA Grade 0) and 156 (47.7%) had LA (LA Grades 1, 2, and 3). Fifteen patients (4.6%) of different types of epilepsy, 178 patients (54.4%) of ischemic stroke, 10 patients (3.1%) of CAHD, 76 patients (23.2%) of different degrees of head and neck vascular stenosis, 135 patients (41.3%) of hypertension, 67 patients (20.5%) of diabetes, 83 patients (25.4%) of hyperlipidemia, 93 patients (28.4%) of smoking history, 84 patients (25.7%) of alcohol consumption history (Table 1).

### 3.2 Single Factor Analysis of LA‐Related Risk Factors

Single factor analysis showed a statistically significant difference in ischemic stroke (*p* = 0.000), CAHD (*p* = 0.007), vascular stenosis (*p* = 0.000), hypertension (*p* = 0.000), age (*p* = 0.000), DBIL (*p* = 0.035), AST (*p* = 0.012), FIB (*p* = 0.009), FT3 (*p* = 0.000), FT4 (*p* = 0.000), CEA (*p* = 0.000), CA199 (*p* = 0.004), SBP (*p* = 0.000) in the LA versus no LA population; while the difference in gender, epilepsy, diabetes, hyperlipidemia, smoking, alcohol consumption, BMI, TC, TBIL, IBIL, ALT, TG, LDL, HDL, HbA1c, FBG, UA, DBP was not statistically significant (Table 1).

### 3.3 Multi‐Factor Analysis of LA‐Related Risk Factors

The age was divided into three groups: < 60 years old, 60–75 years old, and > 75 years old, which were recorded as “Age 0,” “Age 1,” and “Age 2,” respectively. According to the classification of hypertension, SBP is divided into four levels: < 140 mmHg, 140–159 mmHg, 160–179 mmHg, and > 180 mmHg, which are recorded as “SBP 0,” “SBP 1,” “SBP 2,” “SBP 3”. Eighty percent (270 cases) of the total data were randomly selected as the training set, and multifactor analysis was performed in the training set. During this analysis, we briefly tested potential interactions based on prior evidence but only retained those that were statistically significant (“Age × SBP,” “Ischemic stroke × SBP,” “Ischemic stroke × Vascular stenosis,” and “Age × Ischemic Stroke”). This approach helped us focus on the most relevant and clinically meaningful interactions while ensuring the model remained robust and parsimonious. Multicollinearity was evaluated using variance inflation factors (VIFs) for all predictors, including the interaction terms. All VIF values were below 5, suggesting no serious collinearity. We paid special attention to conceptually related variables, such as hypertension and SBP/DBP or diabetes and HbA1c/FBG, but none exceeded accepted thresholds. Each interaction term was selected based on clinical rationale, and was retained only if statistically significant. Coefficients and standard errors remained stable after including these terms, and the final model showed good predictive performance (AUC ≈ 0.90 in the test dataset). Incorporation of the above statistically significant risk factors into the logistic regression model, showed that Age 1 (OR [dominance ratio], 14.315; 95% CI, 6.662 ∼ 30.757; *p* = 0.000), Age 2 (OR, 53.062; 95% CI, 15.661 ∼ 179.783; *p* = 0.000), SBP 1 (OR, 2.927; 95% CI, 1.224 ∼ 7.003; *p* = 0.016), SBP 3 (OR, 15.109; 95% CI, 1.380 ∼ 165.385; *p* = 0.026), ischemic stroke (OR, 5.990; 95% CI, 2.594 ∼ 13.846; *p* = 0.000), FT4 (OR, 4.836; 95% CI, 2.086 ～ 11.216; *p* = 0.000) was statistically significant (Table 2).

**TABLE 2:** Multifactorial regression analysis of leukoaraiosis risk factors.

Variables	Regression coefficients	Standard errors	*p* value	OR value	95% confidence interval
Age 1	2.661	0.390	0.000^*^	14.315	6.662, 30.757
Age 2	3.971	0.623	0.000^*^	53.062	15.661, 179.783
SBP 1	1.074	0.445	0.016^*^	2.927	1.224, 7.003
SBP 2	0.428	0.572	0.454	1.535	0.500, 4.710
SBP 3	2.715	1.221	0.026^*^	15.109	1.380, 165.385
Hypertension	0.574	0.351	0.102	1.774	0.892, 3.531
Ischemic stroke	1.790	0.427	0.000^*^	5.990	2.594, 13.836
Vascular stenosis	−0.078	0.417	0.852	0.925	0.409, 2.095
CAHD	0.025	1.123	0.982	1.025	0.114, 9.257
DBIL	0.546	0.634	0.389	1.727	0.499, 5.977
AST	0.294	0.429	0.493	1.342	0.579, 3.108
FIB	0.408	0.417	0.329	1.503	0.663, 3.407
FT3	0.261	0.551	0.636	1.298	0.441, 3.822
FT4	1.576	0.429	0.000^*^	4.836	2.086, 11.216
CEA	−0.465	0.520	0.371	0.629	0.227, 1.739
CA199	0.861	0.493	0.081	2.366	0.900, 6.218

*Note*: Age 1, 60–75 years old; Age 2, > 75 years old; SBP 1, 140–159 mmHg; SBP 2, 160–179 mmHg; SBP 3, > 180 mmHg.

Abbreviations: AST, aspartate aminotransferase; CA199, glycoconjugate antigen 199; CAHD, coronary atherosclerotic heart disease; CEA, carcinoembryonic antigen; DBIL, direct bilirubin; FIB, fibrinogen; FT3, free triiodothyronine; FT4, free thyroxine; SBP, systolic blood pressure.

* represents p < 0.05, statistically different.

### 3.4 LA Diagnostic Accuracy Evaluation

**FIGURE 3:**
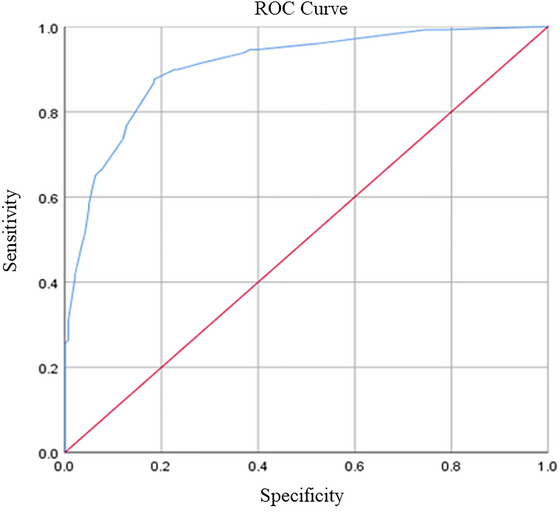
Receiver operating characteristic (ROC) curve for the leukoaraiosis diagnostic model based on age, blood pressure, free T4 hormone, and history of ischemic stroke.

To investigate the sensitivity and specificity of “age, systolic blood pressure, ischemic stroke, and FT4” as diagnostic markers of LA, the specificity (i.e., AUC) for the diagnosis of LA was 0.906 (95% CI 0.870–0.901) as determined by the ROC curve. When the sensitivity is 0.876 and the specificity is 0.184, the optimal critical value is 0.503 (Figure 3).

### 3.5 Sensitivity Prediction of LA Diagnostic Model

The regression coefficients of “Age 1,” “Age 2,” “SBP 1,” “SBP 3,” “ischemic stroke” and “FT4” were multiplied with the remaining 20% of the test set, and the values obtained were used as the criteria for determining the optimal critical values mentioned above to find the sensitivity of the model for LA diagnosis. A sensitivity and specificity of 85.2% were obtained.

## 4 Discussion

In previous studies, age, and hypertension have been shown to be risk factors strongly associated with LA, both in cross‐sectional and prospective trials (Grueter and Schulz 2012; Pantoni and Garcia 2017; Bruno et al. 2023). Aging is probably the most important risk factor for LA (Marek et al. 2018; Grueter and Schulz 2012; Fernando et al. 2006; Park et al. 2007). Although the pathogenesis of LA is not fully understood, its incidence increases with age, especially in people over 60 years of age (Marek et al. 2018). This is consistent with the results of our study. In this study, the age of the population with LA was generally higher than that of the population without LA, and the incidence of LA gradually increased with increasing age. However, it is unclear at what age LA begins, and definitive evidence that it begins at a certain age is lacking (Grueter and Schulz 2012). Most studies have shown that at least more WMLs can be found after the age of 50–65 (Marek et al. 2018; Mijajlović et al. 2011). Our data were divided into three groups of age < 60 years, 60–75 years, and > 75 years, and after correcting the confounding factors, the analysis and comparison revealed that the age group above 75 years was the most important independent risk factor for LA. Research to date have undoubtedly pointed to LA being common in older adults and becoming more prevalent and severe with age.

Our study suggests that age and hypertension are independent risk factors for LA. Therefore, the management of blood pressure level is particularly important to prevent LA. Long‐term hypertension‐induced cerebral small vessel sclerosis and luminal narrowing, resulting in impaired autoregulation of whole brain blood flow, may be one of the main pathogenic mechanisms causing LA (Lin et al. 2017). A longitudinal study by Verhaaren et al. (2013) also found that elevated SBP and DBP were both associated with LA. However, in the results of this study, no significant correlation was seen between LA and DBP, but elevated SBP may be an independent risk factor for LA. We divided SBP into four groups, < 140 mmHg, 140–159 mmHg, 160–179 mmHg, and > 180 mmHg. Further analysis revealed that mild hypertension (i.e., 140–159 mmHg) and severe hypertension (i.e., > 180 mmHg) were highly correlated with LA, and the correlation between severe hypertension and LA was more significant. To date, no study has proposed that SBP has an exact threshold for LA (Grueter and Schulz 2012). Whether the relationship between the two is continuous or has its specific threshold remains to be explored in more longitudinal studies.

LA is more common and more severe in the ischemic stroke population than in the healthy population (Sun et al. 2022; Kongbunkiat et al. 2017; Tang et al. 2021). Most previous cross‐sectional studies suggest that LA is a risk factor for the first onset of stroke and its recurrence (Sun et al. 2022; Debette and Markus 2010). In recent years, there has been increasing evidence that LA is a strong predictor of stroke (Helenius and Henninger 2015; He et al. 2024). One potential mechanism for this association may be due to poor collateral vascular circulation in patients with LA (Iadecola et al. 2023). In our study, ischemic stroke was found to be an independent risk factor for the development of LA, and its effect on LA was second only to that of age and severe SBP. However, lacunar cerebral infarction was excluded from this study, while previous studies have suggested a close association between LA and lacunar cerebral infarction, both of which are manifestations of CSVD and often occur together (Appleton et al. 2020). As to the reason for the association, some authors suggest that lacunar infarction responds to acute injury, whereas LA shows chronic ischemic changes (Grueter and Schulz 2012). These two conditions have different distributions of risk factors, and they may each have different pathological mechanisms, and further studies are needed to elucidate their relevance.

In terms of exploring the relevance of thyroid function to CSVD, the Berlin “Cream & Sugar” substudy by Leonards et al. (2014) found that TSH levels in patients with acute ischemic stroke were independently associated with LA severity. Subsequently, a prospective study by Zhang et al. (2001) found that subclinical hypothyroidism was associated with LA and cerebral microhemorrhage in patients with mild stroke or transient ischemic attack. As a sensitive indicator for thyroid function monitoring, neither of the above two studies mentioned the relationship between LA and FT3 and FT4. Therefore, in this study, two indicators, FT3 and FT4, were collected. We found that there was no statistical difference in FT3, only FT4 was an independent risk factor for LA after correcting for the effect of confounding factors. The reason may be due to the fact that central nervous system, especially oligodendrocytes, are particularly dependent on thyroid hormones during embryonic differentiation, maintenance, and myelin formation. Therefore, changes in thyroid function during the acute phase of ischemic stroke may be a moderator of LA and functional outcome (Schweizer and Köhrle 2013). As for the difference between FT3 and FT4, although FT3 is more active than FT4, all FT4/TT4 in serum is secreted from the thyroid gland. In contrast, only a small percentage (10%–20%) of serum FT3/TT3 is secreted directly from the thyroid gland, and the majority is converted from FT4/TT4 in peripheral blood (Ortiga‐Carvalho et al. 2016). Therefore, strictly speaking, only FT4/TT4 can truly reflect thyroid function. The results of this study are consistent with previous studies, in which only FT4 was an independent risk factor for LA after correcting for the effect of confounding factors (Zhang et al. 2001).

Since the concept of LA was introduced, a series of imaging scales have emerged to diagnose and assess the occurrence and severity of LA. It is difficult to determine which is the most convenient and accurate in terms of diagnostic accuracy and sensitivity. Currently, there are no clinically relevant models to predict the occurrence of LA. In this study, clinical risk factors that may be associated with LA were collected comprehensively, and a diagnostic model based on four indicators, namely age, SBP, ischemic stroke and FT4, was finally established after single factor analysis and multi‐factor analysis correcting for the effects of each confounding factor. Through plotting the ROC curve based on this model, it can be concluded that the model has a high diagnostic efficacy for LA with a diagnostic accuracy of 90.6%. The sensitivity and specificity of using this model to predict LA was 85.2%. Although neuroimaging, such as cranial CT and MRI, is the traditional method for diagnosing LA, its clinical features are gradually gaining attention with the deepening of research on LA in recent years. There is also an urgent need for a quicker, easier, and more sensitive method to screen for and predict the onset and progression of LA at an early stage in the clinic. In light of our findings, the high diagnostic accuracy demonstrated by our ROC analysis confirms that our model can reliably distinguish between individuals with and without LA at an early stage. By incorporating commonly available clinical parameters—age, SBP, ischemic stroke history, and FT4 levels—the model provides a cost‐effective and accessible alternative that complements conventional neuroimaging techniques, especially in settings where immediate access to CT or MRI is limited. Importantly, this model can be integrated into clinical workflows as a supportive tool rather than a replacement for imaging. First, it can act as an early‐warning and triage system in primary care or resource‐limited hospitals, helping clinicians prioritize high‐risk patients for timely imaging and intervention. Second, it can serve as an additional layer of information during routine visits, functioning like a simple risk score that guides decision‐making and can be embedded in electronic health records. Third, the model can be recalculated during follow‐up visits, providing a quantitative measure to signal when repeat imaging may be warranted. While neuroimaging remains the definitive diagnostic approach, our model serves as a valuable adjunct for preliminary screening, risk stratification, and dynamic monitoring, ultimately facilitating timely intervention and improving clinical management.

In our analysis, some variables which are seen as potential risk factors of LA did not reach statistical significance, such as BMI, smoking, and alcohol consumption. The result may reflect the limited range of these factors within our study population or the stronger influence of other predictors. For BMI, a relatively narrow range and the dominance of other vascular risk factors may have diminished its measurable impact. Smoking, although recognized as a cerebrovascular risk factor, may not have been represented at sufficient intensity or duration to show a detectable effect, and the sample size may have lacked the power to identify a modest association. Similarly, alcohol consumption, defined by a specific threshold in our study, might have had a more complex or indirect relationship that was not fully captured. While these factors remain relevant to overall health, their lack of significance here suggests that their roles in this context are less direct, helping us focus on more impactful predictors and refine our conclusions.

Existing research indicates that ethnic differences can influence the development and progression of LA through multiple dimensions, including genetic susceptibility, epigenetic regulation, environmental exposures, lifestyle factors, and access to healthcare (Sun et al. 2022). For instance, variations in hypertension‐related genes (such as the ACE I/D polymorphism and AGT gene distributions) may predispose certain ethnic groups to more severe vascular damage (Mo et al. 2024; Huang et al. 2019), while differences in DNA methylation patterns could further modulate LA risk (Huang et al. 2018). In addition, disparities in blood pressure control, dietary habits (such as high salt intake in some Asian populations), and regional climatic factors have been shown to influence LA prevalence and progression (Hannawi 2024). Our study sample is primarily drawn from Western China, a region characterized by unique genetic, cultural, environmental, and socioeconomic backgrounds. Several factors specific to this region may increase LA risk. First, genetic predispositions such as the MTHFR C677T polymorphism, highly prevalent in Han Chinese, can elevate homocysteine levels and are associated with more severe WMLs (Rutten‐Jacobs et al. 2016). Variations in other hypertension‐related genes may also increase susceptibility to microvascular damage (Liu et al. 2020). Second, lifestyle patterns in Western China, including high salt intake from preserved foods and salted butter tea, higher smoking prevalence among men, and limited healthcare access in rural areas, may accelerate hypertension and vascular injury (Jiang et al. 2023). Third, environmental exposures unique to this region, such as chronic hypoxia from high‐altitude living, indoor air pollution from coal or wood burning, and extreme climate conditions, can further impair cerebral small vessels (Yao et al. 2025). Although we adjusted for major confounders such as age, sex, and blood pressure control, unmeasured factors like diet, cultural habits, healthcare access, and environmental exposures may still have influenced our findings. For example, uniformly high salt intake could amplify baseline hypertension, making it easier to detect associations with LA but masking the effects of other variables. Similarly, delayed diagnosis due to limited healthcare resources might have skewed our sample toward more advanced disease stages. These residual confounders may reduce the external validity of our results. Despite these limitations, our study design and methods provide strong internal validity. To enhance generalizability, future studies should include multi‐ethnic, multi‐regional cohorts and prospectively collect detailed data on dietary, environmental, and socioeconomic factors. Incorporating genetic testing across populations will further clarify how ethnicity and geography interact with vascular risk to influence LA. Such steps will help validate and extend our findings to more diverse populations.

In our study, based on a comprehensive collection of clinical data, the four indicators most associated with LA‐age, SBP, ischemic stroke, and FT4‐were analyzed. We utilized a model to assess their accuracy, sensitivity, and specificity in predicting LA. Moreover, these four indicators are readily available in clinical work, with rapid and easy assessment and high reproducibility. It offers the possibility of initial screening of LA‐prone populations. However, this study also has some limitations.

First, our sample size is modest and drawn exclusively from tertiary neurology inpatients, which may not represent the broader population. Neurology wards enrich the LA spectrum—as reflected by our internal audit (∼60% LA among unique admissions with MRI)—whereas community‐based samples at similar mean ages typically show substantially lower prevalence (Huang et al. 2024). Consequently, selection of a tertiary inpatient cohort can introduce severity‐spectrum, referral, and indication biases that are likely to inflate absolute prevalence and absolute risk estimates relative to the general population. By contrast, the direction of associations between key exposures and LA is expected to be preserved across settings, with potential attenuation of effect sizes in community cohorts due to a lower burden of vascular comorbidity. To address these issues in line with STROBE guidance, we have (i) explicitly stated the likely direction of bias and its qualitative magnitude (using inpatient–community contrasts as context), and (ii) outlined mitigation and validation steps. Specifically, we are initiating a prospective, multicenter validation that includes neurology and physical examination centers to capture a healthier, ambulatory case‐mix. We will standardize imaging and clinical data collection and assess transportability across strata of age and vascular risk. Where feasible, we will incorporate design and analysis strategies to further evaluate robustness.

Second, this study is a cross‐sectional study, which can only show the correlation between LA and clinical indicators, but it cannot explain the causal relationship between them. To address these limitations and strengthen the clinical applicability of our model, we have planned a prospective, multi‐center validation study. This study will involve a larger and more diverse cohort across different regions to enhance the generalizability of our findings. By collecting real‐time clinical and imaging data over time, we aim to evaluate the model's predictive accuracy and calibration in real‐world settings. The study will also allow us to refine and optimize the model if necessary, ensuring its robustness across varied patient populations. We are actively collaborating with neurology and physical examination centers to standardize data collection protocols and track patient outcomes longitudinally. These efforts will ultimately contribute to the clinical translation of our model and improve its applicability in routine practice.

Third, there may be other potential factors that were not included in this study, either due to their lesser frequency in the literature or practical limitations in data collection, which might contribute to the development of LA. For example, we considered including certain inflammatory markers such as interleukin‐6 (IL‐6) and estimated glomerular filtration rate (eGFR) but ultimately excluded them. Although some studies have noted associations between these factors and LA, the evidence has not been consistently replicated, and these parameters were not reliably measured for all patients in our multicenter dataset (for instance, inter‐center differences in laboratory assays could skew eGFR values) (Leonardo and Fregni 2023; Wei et al. 2022). Similarly, formal cognitive test scores and sleep apnea indicators were omitted due to inconsistent data availability. We did consider proxy measures (such as educational attainment as a surrogate for cognitive function or screening questionnaires for sleep apnea), but these proxies were too imprecise and inconsistently recorded to include without introducing bias (Huang et al. 2024). Therefore, our model focuses on common clinical factors that are readily obtainable, ensuring the model remains practical and broadly applicable. We acknowledge that excluding these specialized factors is a trade‐off that might overlook some contributions to LA risk, but it was necessary to maintain feasibility and generalizability.

Finally, external validation on an independent cohort was not performed at this stage. We prioritized internal validation to evaluate the model's effectiveness and predictive capability, using 80% of the dataset for training and 20% for testing. The results demonstrated high sensitivity, specificity, and AUC, with consistency between training and testing sets, suggesting good generalizability and no significant overfitting. Building on these encouraging findings, we plan to extend validation to independent cohorts in the future. At present, no formal pilot studies have been initiated, but preliminary discussions with hospitals in different regions of China—including both tertiary neurology departments and physical examination centers—are ongoing. Hospital selection will be guided by patient volume, availability of neuroimaging facilities, and the ability to follow standardized protocols. To ensure consistency across centers, we are developing a unified framework for imaging, clinical variables, and data preprocessing. These preparatory steps will lay the groundwork for rigorous multicenter external validation and contribute to the clinical translation of our model.

## 5 Conclusions

Among LA patients, we found that factors affecting the development of the disease include age, SBP, ischemic stroke, and FT4 level. The model of “age, systolic blood pressure, ischemic stroke, FT4” have relatively ideal sensitivity and specificity in predicting the development of LA.

## Author Contributions

**Lingqi Sun**: statistical analysis, writing – original draft, writing – review and editing. **Wang Guo**: statistical analysis, writing – original draft, writing – review and editing. **Yi Li**: writing – original draft. **Li Fang**: writing – review and editing. **Ji Ma**: conceptualization, writing – review and editing. **Xiangdong Tang**: conceptualization, supervision. All authors take responsibility for the integrity and the accuracy of this manuscript.

## Ethics Statement

The protocol of this study was approved by Ethics Committee of Air Force Hospital of the Western Theater of the Chinese People's Liberation Army. All methods were performed in accordance with the Declaration of Helsinki.

## Consent

The need for informed consent was waived by Ethics Committee of Air Force Hospital of the Western Theater of the Chinese People's Liberation Army.

## Conflicts of Interest

The authors declare no conflicts of interest.

## Peer Review

The peer review history for this article is available at https://publons.com/publon/10.1002/brb3.71006.

## Data Availability

The datasets used and/or analyzed during the current study available from the corresponding author on reasonable request.
